# Trans-synaptic spreading of alpha-synuclein pathology through sensory afferents leads to sensory nerve degeneration and neuropathic pain

**DOI:** 10.1186/s40478-021-01131-8

**Published:** 2021-02-25

**Authors:** Nelson Ferreira, Nádia Pereira Gonçalves, Asad Jan, Nanna Møller Jensen, Amelia van der Laan, Simin Mohseni, Christian Bjerggaard Vægter, Poul Henning Jensen

**Affiliations:** 1 Danish Research Institute of Translational Neuroscience (DANDRITE), Nordic EMBL Partnership for Molecular Medicine, Department of Biomedicine, Aarhus University, 8000 Aarhus C, Denmark; 2 International Diabetic Neuropathy Consortium (IDNC), Aarhus University Hospital, Aarhus N, Denmark; 3 Department of Biomedical and Clinical Sciences, Linköping University, Linköping, Sweden

**Keywords:** Alpha-synuclein, Parkinson’s disease, Neuropathic pain, Nociception, Protein aggregation

## Abstract

Pain is a common non-motor symptom of Parkinson’s disease (PD), with current limited knowledge of its pathophysiology. Here, we show that peripheral inoculation of mouse alpha-synuclein (α-Syn) pre-formed fibrils, in a transgenic mouse model of PD, elicited retrograde trans-synaptic spreading of α-Syn pathology (pSer129) across sensory neurons and dorsal nerve roots, reaching central pain processing regions, including the spinal dorsal horn and the projections of the anterolateral system in the central nervous system (CNS). Pathological peripheral to CNS propagation of α-Syn aggregates along interconnected neuronal populations within sensory afferents, was concomitant with impaired nociceptive response, reflected by mechanical allodynia, reduced nerve conduction velocities (sensory and motor) and degeneration of small- and medium-sized myelinated fibers. Our findings show a link between the transneuronal propagation of α-Syn pathology with sensory neuron dysfunction and neuropathic impairment, suggesting promising avenues of investigation into the mechanisms underlying pain in PD.

## Introduction

Parkinson’s disease (PD) is traditionally considered a neurodegenerative motor disorder, but it is becoming increasingly clear that non-motor symptoms also adversely impact the quality of life for PD patients [48]. A significant number of PD patients (30–85%) reportedly suffer from some form of acute or chronic pain, including musculoskeletal, fluctuation related, central, nocturnal, orofacial and peripheral pain [48]. Some PD-associated dystonia-related musculoskeletal pain may respond to dopaminergic medication [24]; otherwise, deep brain stimulation of the subthalamic nucleus has evidenced a reduction in the number of body areas experiencing pain and has shown to improve pain scores from late-stage PD patients [29]. It has been suggested that pain sensations may precede the onset of motor symptoms of PD by several years [24]. Moreover, there is no direct correlation between motor impairment and altered pain thresholds, indicating that motor dysfunction and pain may represent different pathophysiological processes in the progression of PD. Whether nociceptive processing is impaired in the early phases of PD, before the onset of motor symptoms, remains largely unexplored.

Although the pathophysiology of pain in PD remains poorly understood [25], clinical examination of some PD patients demonstrate a significant decrease in tactile and thermal thresholds together with a reduction in mechanical pain perception and a significant loss of epidermal nerve fibers and Meissner corpuscles, independent of patient age or disease duration [41]. These findings suggest that changes in receptor size and peripheral deafferentation of hitherto unknown etiology could play a key role in sensory dysfunction of PD patients [40] and be in accordance with α-Syn-dependent pathophysiology in peripheral nerve fibers [17, 62]. Recently, it has been suggested that α-Syn misfolding may begin in peripheral nerves and spread in a prion-like fashion to the central nervous system (CNS), leading to PD pathology [3, 44, 47, 54]. Indeed, PD pathology can be induced in the brain and spinal cord of α-Syn transgenic mice expressing mutant A53T human α-Syn (M83 line) by a single peripheral intramuscular injection of α-Syn preformed fibrils (PFF) [47]. This increased face-value of the M83 prion-like model prompt us to investigate if these animals may develop neuropathic pain that is a common non-motor PD symptom.

In the present study, we show that mouse α-Syn PFF injected peripherally in the gastrocnemius muscle of the M83 PD mouse model are able to spread and induce α-Syn inclusion pathology alongside neuroanatomical sensory connections, causing degeneration of sensory pathways as well as nociceptive hypersensitivity. We hypothesize that intraneuronal aggregation of α-Syn might elicit dysfunction in peripheral sensory neurons, inducing plastic changes in central pain processing pathways that are ultimately integrated into painful sensations.

## Materials and methods

### Production and purification of α-Syn

The expression vector for mouse α-Syn was a kind gift from Dr. Virginia M. Lee. It was expressed in BL21(DE3) competent cells and purified as previously described [36]. Briefly, α-Syn purification involved dialysis of proteins against 20 mM Tris pH 6.5 overnight, followed by ion-exchange chromatography on a Poros HQ50 column (Thermo Fisher Scientific) with a 0–2 M NaCl gradient. This method was followed by an additional reverse phase-high pressure liquid chromatography purification step on a Jupiter C18 column (Phenomenex, Torrance, CA) in 0.1% trifluoroacetic acid with an 0–90% acetonitrile gradient. Isolated α-Syn was then extensively dialysed against PBS pH 7.4 overnight followed by an additional dialysis step against 20 mM ammonium bicarbonate overnight. Protein concentration was determined by bicinchoninic acid (BCA) protein concentration assay (Pierce). The proteins were subsequently aliquoted, lyophilized, and stored at − 80 °C until use.

### Quantitative α-Syn fibril assembly and sedimentation

Soluble monomeric mouse α-Syn (5 mg/ml) was assembled into preformed fibrils (PFF) by incubation at 37 °C in phosphate-buffered saline (PBS) pH 7.4 (PBS, Gibco) with continuous shaking at 1050 r.p.m. (Eppendorf Thermotop). The generated PFF were harvested by centrifugation at 15,600 g 25 °C for 30 min, and then resuspended in PBS at a concentration of 2 mg/mL, as determined by BCA. Subsequently, PFF were sonicated for 20 min using a Branson 250 Sonifier at 30% intensity, before being aliquoted and frozen at − 80 °C until further use. A fraction of each PFF batch was set aside for SDS-PAGE, (trans,trans)-1-bromo-2,5-bis-(4-hydroxy)styrylbenzene (K114) fluorometry and dynamic light scattering (DLS) analysis of purity, amyloid structure and particle size.

### SDS-PAGE

SDS sample buffer (4% SDS, 40% glycerol, 1% bromophenol blue, 50 mM Tris, pH 6.8) was added to mouse PFF, which were heated to 96 °C for 15 min and analysed by SDS-PAGE (sodium dodecyl sulphate–polyacrylamide gel electrophoresis). The Coomassie blue stained SDS-PAGE gels were scanned using Image J software (National Institutes of Health, Bethesda, MD, USA) for quantification.

### K114 fluorometry

To quantify the amount of amyloid formation, samples were monitored by (trans,trans)-1-bromo-2,5-bis-(4-hydroxy)styrylbenzene (K114) fluorometry as described previously [12]. In brief, samples were analysed by incubating a fraction of each sample with the K114 (50 µM) in 100 mM glycine, pH 8.5, and measuring fluorescence (λ_ex_ = 380 nm, λ_em_ = 550 nm, cutoff = 530 nm) with an EnSpire 2300 Multilabel Reader (Perkin Elmer).

### Dynamic light scattering (DLS)

PFF (2 mg/ml) were subjected to ultrasound breakage for 20 min using a Sonifier (Branson 250; 30% Duty Cycle) equipped with a water jacket cooling system to avoid sample overheating. Then, the size distribution profile of PFF in suspension was measured by DLS using a Wyatt DynaPro NanoStar instrument at 25 °C. Data was processed using the Dynamics 7.5.0.17 software package, with the solvent (PBS) background signal subtracted from each sample.

### Seeding test of PFF in organotypic hippocampal slice cultures

Organotypic hippocampal culture slices (OHCS) were created from C57BL/6J pups on post-natal day 7 according to Stoppini et al. [51]. In order to validate the ability of the mouse full-length (1-140) α-Syn PFF for seeding aggregation before using them for in vivo experiments, the PFF were injected into the dentate gyrus of the OHCS, as previously described [18]. Slices were fixed 7 days post injection (dpi), and stained for pathological aggregates using conformation-specific α-Syn antibody MJF-14 (rabbit mAb MJF-14-6-4-2, 1:25,000, Abcam #ab209538) and pSer129 (mouse mAb 11A5, 1:10,000, kindly provided by ImagoPharmaceuticals), as described previously [18]. Alexa Fluor 488 anti-rabbit and Alexa Fluor 568 anti-mouse (Invitrogen, #A11008 and #A11004, 1:2000) were used for detection, along with 4′,6-diamidino-2-phenylindole (DAPI, TH.GEYER, 5 µg/mL) for staining nuclei. As negative controls, C57BL/6J OHCS were injected with either sterile PBS or monomeric α-Syn and processed as above. Furthermore, α-Syn knockout OHCS were injected with human S129A PFF and processed as above.

### Animal model

Experimental procedures involving mice were approved by The Danish Animal Experiments Inspectorate (license 2017-15-0201-01203) and followed the Danish and European Animal experimentation guidelines and legislations (directive 2010/63/EU).

Mice were housed in a temperature-controlled room under a 12 h light/dark period with water and food ad libitum. Three-month-old heterozygous female mice transgenic for human mutant A53T α-Syn (M83^+/−^) were bilaterally injected with vehicle (PBS pH 7.4, *n* = 6), monomeric α-Syn (2 × 5 μL at 2 mg/mL, *n* = 8) or full-length (1-140) mouse α-Syn PFF (2 × 5 μL at 2 mg/ml, *n* = 8). An additional control experiment was performed where mice were injected with full-length (1-140) mouse α-Syn PFF (*n* = 4) in the right hindlimb (ipsilateral) and vehicle in the left hindlimb (PBS, pH 7.4, contralateral). Mice were anaesthetised with isoflurane (3.5%) inhalation. Intramuscular injections were made using different 10-μL Hamilton syringes with a 25-gauge needle to avoid any cross-contamination and performed by inserting a needle ∼1 mm deep into the gastrocnemius muscle, as described elsewhere [47]. Hindlimb clasping behaviour was monitored and scored regularly as previously reported [23, 27]. Briefly, the mouse is lifted by its tail, and the time the hindlimbs are retracted towards the abdomen is scored on a 4-point scale, from 0 to 3. A score of 0 is given if the hindlimbs are continuously spread away from the abdomen; a score of 1 when one hindlimb is retracted towards the abdomen more than 50% of the suspended time; and score of 2 if both hindlimbs are partially retracted for 50% of the time. A maximum score of 3 implies that both hindlimbs were completely retracted and touching the abdomen for more than 5 s of the suspended time [23, 27].

At 45 dpi, while motor function remains intact and before the development of debilitating motor impairments [47], M83 mice were euthanized with an overdose of isoflurane and perfused with PBS pH 7.4 with phosphatase inhibitors (25 mM β-glycerolphosphate, 5 mM NaF, 1 mM Na_3_VO_4_, 10 mM Na-pyrophosphate) before dissecting brain, spinal cord, dorsal roots and lumbar (L3-L5) dorsal root ganglia (DRG). Tissues were snap frozen and stored at − 80 °C until use, or fixed and processed as described below.

### Evaluation of mechanical allodynia

Mice were acclimatized in a Plexiglas cage atop a mesh metal grid for approximately 30 min prior to testing. The “ascending stimulus” method was used to determine the mechanical withdrawal thresholds, by manually applying calibrated Semmes–Weinstein monofilaments (Stoelting Co) into the plantar surface of the hind paws [14]. A response is considered positive if the mouse exhibits any nocifensive behaviors, including sudden paw withdrawal, licking, flinching or trembling of the paw, either while applying the stimulus or immediately afterwards [13]. A total of five stimuli per filament were recorded and when at least three out of five trials rendered a positive response, the corresponding force was defined as the tactical threshold (expressed in grams) [46]. The average of both paws per mouse was used for the statistical analysis.

### Nerve conduction velocity recordings

Mice were anesthetized with 2% isoflurane for measuring sural sensory and sciatic motor nerve conduction velocities (NCV), using a Viking Quest apparatus (Natus Neurology Incorporated, USA). For recording sural sensory nerve conduction velocity (SNCV), electrodes were placed in the dorsum of the hind paw with supramaximal stimulation at the ankle.

For motor nerve conduction velocity (MNCV), the stimulation needle electrodes (Natus Biomedical, Madison, WI) were set at the ankle with recording electrodes placed about 7–8 mm away from it, in the dorsum of the hind paw. A second measurement was performed with the stimulation set at the sciatic nerve notch. Sciatic-tibial MNCV was then calculated following the formula: [MNCV (m/s) = D/L], where (L) represents the take-off latency (ms) of the sciatic nerve and (D) the distance between the stimulating and recording electrodes (mm). After the electrophysiological recordings, mice were terminally anaesthetized with an overdose of isoflurane for tissue dissection.

### Western blot

L3-L5 DRG were homogenised and analysed by Western blot as previously described [31, 32]. Briefly, samples were homogenised in lysis buffer (approx. 10 weight/volume ratio; 20 mM Tris pH 7.4, 0.32 M sucrose, 5 mM EDTA and 1 cOmplete™ proteinase inhibitor tablet/10 mL (Roche), 25 mM sodium fluoride, 1 mM sodium orthovanadate, 10 mM sodium pyrophosphate). Homogenates were then centrifuged at 25,000× *g* for 30 min at 4 °C. The resulting supernatant was saved as the whole-tissue homogenate. Protein concentration was determined by BCA (Sigma, MO, USA). Whole-tissue homogenate (20 μg protein) was dissolved in loading buffer (100 mM Tris–HCl, 8% SDS, 24% glycerol, 0.02% bromophenol blue, pH 6.8) and the samples were then denatured at 95 °C for 10 min. After centrifugation for 5 min at 25,000× *g*, the supernatant was loaded into 16% Tricine gels (Novex) or 8–16% polyacrylamide gel (GenScript). Proteins were blotted into PVDF membranes using iBlot® 2 Dry Blotting System (Thermo Fischer). The membranes were then fixed with 4% paraformaldehyde (PFA) in PBS for 30 min; then boiled in PBS for 5 min. After being blocked for 1 h (TBS, 0.01% Tween, skimmed milk powder, pH 7.6), membranes were incubated with primary antibodies, mouse mAb pSer129-α-Syn (11A5, 1:2,000), mouse Syn-1 (BD Biosciences #610787, 1:1,000), mouse anti-actin (Sigma A5441, 1:5000), or mouse anti-β-III tubulin (Sigma, T5076, 1:5000), ON at 4 °C, and subsequently incubated with secondary HRP conjugated mouse immunoglobulins (Dako, Denmark) for 1.5 h at RT. Protein bands were visualised with ECL® (GE Healthcare, UK) and image acquisition performed with Fuji LAS-3000 intelligent dark box (Fujifilm, Japan).

### Immunofluorescence

#### Immunofluorescence for frozen DRG sections

Dorsal roots and L4 DRG (*n* = 4 per group) were fixed in 4% paraformaldehyde (PFA) overnight before being transferred to the 30% sucrose cryoprotection solution, where they stayed overnight at 4 °C. Tissue was then embedded in Tissue-Tek (Sakura) and snap frozen in isopentane on dry ice. Longitudinal sections were cut at 10 μm in a cryostat. After drying, tissue sections were incubated in 20% methanol for 5 min, blocked with a buffer containing 10% fetal bovine serum in 0.3% Triton X-100 in PBS for 1 h at room temperature, followed by overnight incubation with the primary antibodies against mouse mAb pSer129-α-Syn (11A5, 1:5,000) and neurofilament M (NF-M) (Millipore AB1987, 1:200) at 4 °C. Alexa Fluor 488 anti-mouse and Alexa Fluor 568 anti-rabbit-labelled secondary antibodies (Molecular Probes, A10037 and A21206 respectively, 1:500) were incubated for 2 h and Hoechst (Sigma, 1:10,000) was used to label the nuclei, visualized in blue. Slides were mounted with DAKO fluorescent mounting medium and Z-stacked images captured by confocal microscopy (LSM780, Carl Zeiss, Germany).

#### Immunohistochemistry of paraffin spinal cord and brain sections

Immunohistochemistry on 10 µm thick sections of formalin fixed paraffin embedded spinal cord and brain was performed after deparaffinization and antigen retrieval in citrate buffer pH 6. The following primary antibodies were employed: pSer129-α-Syn (D1R1R, Cell Signaling #23706, 1:500 or mouse mAb 11A5 1:1000). Neuronal nuclei marker (NeuN, Millipore, MAB377, 1:500), and glial fibrillary acidic protein (GFAP, Cell Signaling #12389, 1:200). Alexa-Fluor fluorophore conjugated secondary antibodies (Thermo Fisher) were used (1:1000) for primary antibody detection. High resolution panoramic views were obtained using Olympus VS120 digital slide scanner (equipped with single-band emitters for Hoechst, FITC, Cy3, Cy5 and Cy7), and 20 X views were extracted using OlyVia software (Olympus). Alternatively, sections were imaged using Zeiss observer inverted microscope equipped with colibri 7 LED illumination operated using ZenPro software (Zeiss).

### Transmission electron microscopy (TEM)

Dorsal roots (vehicle *n* = 6, mouse monomeric α-Syn *n* = 8 and mouse α-Syn PFF *n* = 8) were dissected, immediately post fixed for 1 h in 2% osmium tetroxide (OsO4) and stained with 2% uranyl acetate in 50% ethanol. Dehydration was performed in a series of ascending concentrations of ethanol and pure acetone (100%). Samples were then embedded in Epoxy (48 h at 60 °C) using Embedding Medium Kit (Sigma-Aldrich, Sweden AB), after a three-step infiltration in a mixture of acetone embedding medium (embedding medium: acetone, 1:3, 1:1, 3:1).

Leica UC7 ultra microtome (Leica Microsystems GmbH, Vienna, Austria) was used for taking 60-nm thick sections that were then collected onto formvar-coated copper slot grids and counterstained with uranyl acetate and lead citrate. A 100 kV transmission electron microscope (JEM 1230, JEOL Ltd., Tokyo, Japan) was used for sample observation.

## Results

### Characterization of α-Syn PFF and trans-synaptic prion-like propagation in vitro

**Fig. 1:**
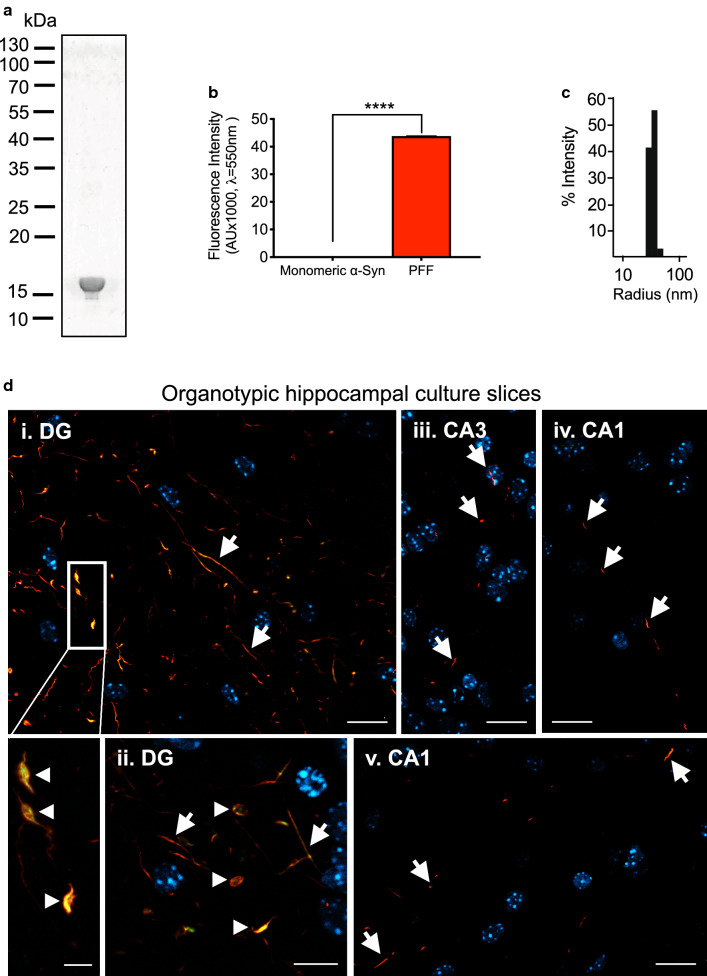
PFF induce α-Syn aggregation and prion-like propagation in organotypic hippocampal slices. Full-length (1-140) mouse PFF were characterized using diverse biochemical and biophysical methodologies. **a** Coomassie blue staining after SDS-PAGE protein separation. **b** K114 fluorescent amyloid assay. Y-axis demonstrates the K114 fluorescence in arbitrary units. Data are shown as mean ± SEM, ****P < 0.0001 based on two-tailed unpaired t test. **c** Dynamic light scattering (DLS) analysis. **d** Following in vitro characterization and validation, PFF were injected into an organotypic hippocampal culture slice (OHCS) model. Abundant aggregation, especially at the dentate gyrus (DG), is seen, 7 days post injection (dpi) of mouse PFF, in mouse OHCS. Aggregation is observed both at the injection site (DG) and spreading throughout the hippocampal slice to the CA3 and CA1 region. Aggregates are positive for the conformation-specific MJF-14 epitope (green) and for serine-129 phosphorylation (red, antibody 11A5). Scale bars: i, iii–v: 20 µm, inset: 5 µm, ii: 10 µm

In order to validate our preparation of sonicated mouse α-Syn PFF prior to inoculation in mice, we analysed PFF purity, size and seeding capacity (Fig. 1). Coomassie blue SDS-PAGE staining showed a single 17 kDa protein band corresponding to α-Syn monomer (Fig. 1a), thus confirming the high purity (> 99%) of the PFF used for this study. For monitoring beta-sheet containing amyloid formation as a measure of α-Syn aggregation, we performed K114 amyloid fluorometry and used the monomeric α-Syn starting material as the negative control (Fig. 1b). As expected, a dramatic increase in fluorescence was observed in the presence of PFF compared to α-Syn monomers (Fig. 1b). The particle size of PFF is of paramount importance for their pathogenicity. It has been shown that sonicated α-Syn PFF with an average size of ~ 50 nm hydrodynamic radius (Rh) or smaller induce the most pathology in vitro and in rodent models of disease [1, 52]. Our dynamic light scattering (DLS) results show that our PFF have an average Rh of 38.8 nm which is below the recommended length cut-off for α-Syn aggregate seeds [1, 52] (Fig. 1c).

The evaluation of the templated seeding capacity of PFF is a crucial step in validating a PFF batch before setting up a resource-intensive in vivo study. Therefore, to determine the templating activity of our PFF preparation, we injected them into the dentate gyrus (DG) of organotypic hippocampal culture slices (OHCS), as shown previously [18]. Injection of PFF in OHCS resulted in the formation of short, serpentine aggregates, formed by the recruitment of endogenous α-Syn at the injection site in the DG, as detected by conformation-specific antibody MJF-14 and pSer129-antibody 11A5 (Fig. 1d). Apart from templating aggregation at the site immediately exposed to exogenous PFF, the injection also triggered a subsequent spreading of aggregation throughout the connected regions of the hippocampal formation, namely cornu ammonis (CA) 3 and 1 (Fig. 1d), thus confirming the ability of the PFF to propagate in a trans-synaptic prion-like fashion ex vivo. The validity of the used antibodies as markers for aggregate pathology was corroborated by the lack of staining in slices injected with either sterile PBS or monomeric α-Syn (Additional file 1: Supplementary Fig. 1a, b). Furthermore, α-Syn knockout (ASKO) slices injected with PFF produced no aggregate staining (Additional file 1: Supplementary Fig. 1c), proving that the aggregates in Fig. 1d are indeed templated, endogenous α-Syn pathology. Overall, these results confirm the sonicated PFF are pure amyloid α-Syn particles of a homogenous size capable of inducing trans-synaptic spreading of aggregate pathology in brain tissue.

### Evaluation of the mouse model sensorimotor phenotype

**Fig. 2:**
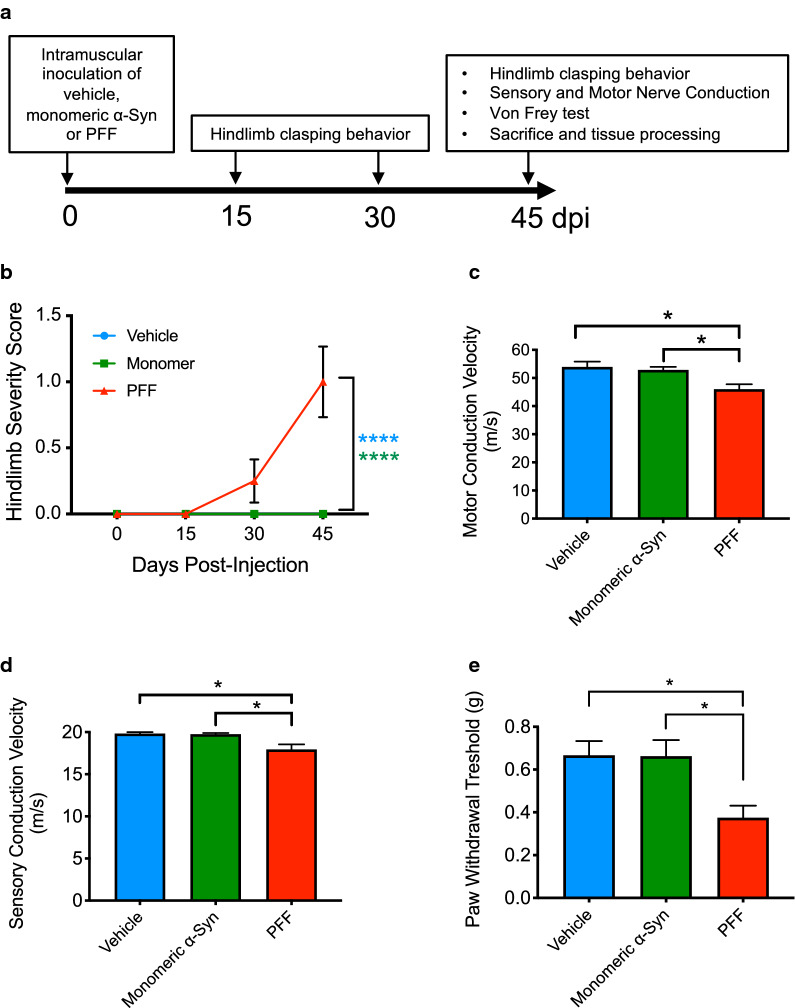
Pathological α-Syn induce nociceptive impairment. **a** Schematic illustration of the experimental design. Three-month-old M83^+/−^ were bilaterally injected with vehicle (PBS, pH 7.4, *n* = 6), mouse monomeric α-Syn (2 × 5 μl at 2 mg/ml, *n* = 8) or mouse α-Syn PFF (2 × 5 μL at 2 mg/ml, *n* = 8), by intramuscular injection into the gastrocnemius muscle. Hindlimb clasping behaviour was monitored and scored regularly. At 45 dpi, mice were tested for hindlimb clasping, sensory and motor nerve conduction and plantar Von Frey filaments, before being euthanized. Tissues, including brain, spinal cord, dorsal roots and lumbar (L3–L5) dorsal root ganglia (DRG) were then snap frozen and stored at − 80 °C until use, or fixed and processed for immunohistochemistry. **b** Hindlimb clasping was scored on a scale from 0 to 3 as a function of dpi and displayed as mean ± SEM. The PBS- and monomeric α-Syn-injected mice did not develop clasping. Results shown as mean ± SEM, as determined by two-way ANOVA followed by Tukey’s multiple comparisons test. ****P < 0.0001. At 45 dpi mice were tested for **c** Motor nerve conduction velocity. Error bar indicate mean ± SEM as determined by one-way ANOVA followed by Tukey’s multiple comparison test. *P < 0.05. **d** Sensory nerve conduction velocity. Results shown as mean ± SEM as determined by one-way ANOVA followed by Tukey’s multiple comparison test. *P < 0.05. **e** Mechanical allodynia using the Von Frey test. Error bars indicate mean ± SEM as determined by one-way ANOVA followed by Tukey’s multiple comparison test. *P < 0.05

To determine if peripherally administered α-Syn PFF in the hind limb gastrocnemius muscle modulate nociceptive pain responses during their templated spreading from the peripheral nervous system to the brain, we used M83^+/−^ mice as they do not spontaneously develop disease within the time frame of our experiment (Fig. 2a, up to 45 dpi) [47].

Hindlimb clasping reflex towards the abdomen is a known marker of neurodegeneration in different mouse models of neurodegenerative diseases [34, 35], including Parkinson’s disease [19]. Our results show that mice injected with vehicle (PBS, pH 7.4) or monomeric α-Syn did not present any signs of hindlimb clasping within the 45 dpi period of observation (Fig. 2b). In contrast, the PFF cohort displayed progressive clasping that became evident at 30 dpi (2 mice out of 8 mice clasping, mean score of 0.25) and after 45 dpi amounted to 6 out of 8 mice clasping with a mean score of 1.0 (Fig. 2b).

A decay in peripheral nerve function has been associated with PD [10]. Therefore, electrophysiological examination can provide valuable information on neurofunctional abnormalities allowing for a better recognition of peripheral nerve degeneration. Our results show that, at functional level, peripherally injected PFF mice showed impairments in both motor (13% reduction, Fig. 2c) and sensory (9% reduction, Fig. 2d) nerve conduction velocities.

To investigate whether the observed functional nerve damage translate into hypersensitivity (mechanical allodynia), similarly to what is observed in other cases of PNS injury [20, 46], we tested pain behavior with Von Frey filaments of varying forces. Our results show that injection of PFF, but not vehicle or monomeric α-Syn, elicited mechanical allodynia by reducing the paw withdrawal threshold by 44% (Fig. 2e).

### Trans-synaptic propagation of α-Syn pathology from the periphery into central nociceptive pathways

**Fig. 3:**
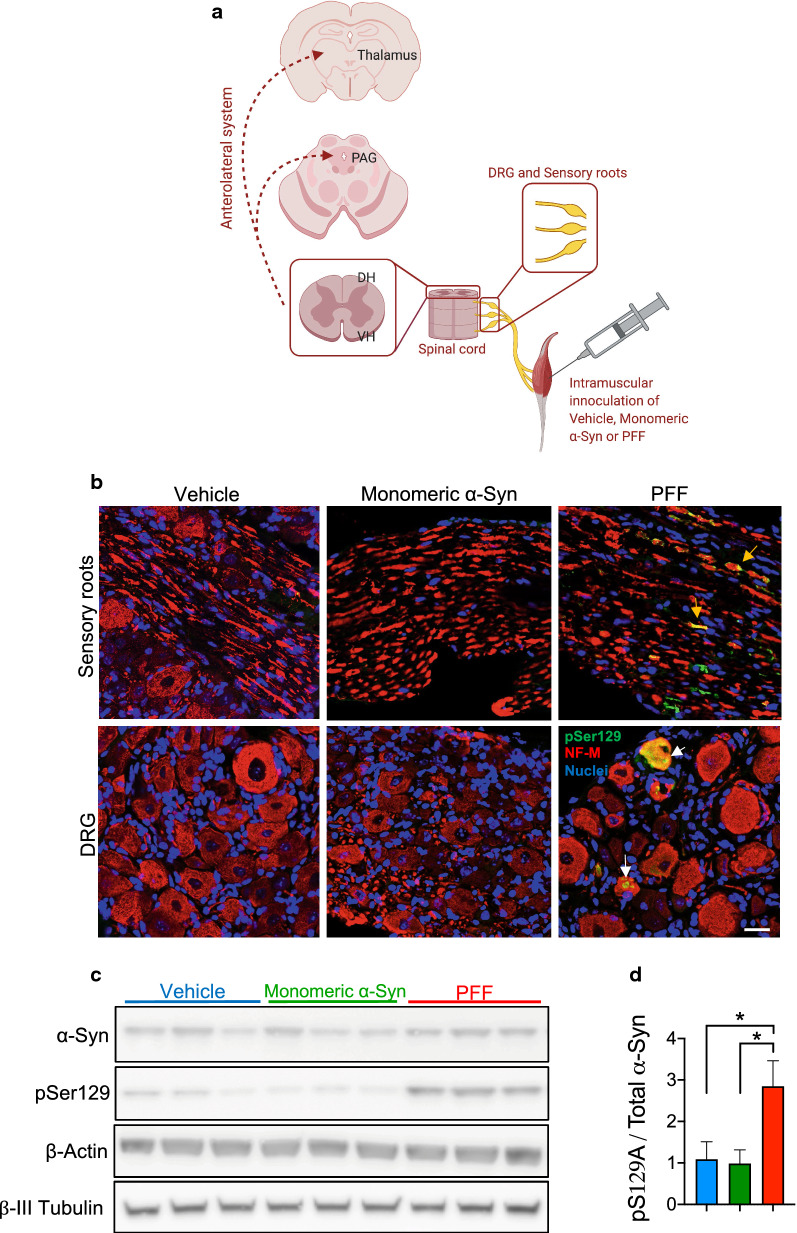
Peripherally injected PFF lead to prion-like α-Syn aggregation in dorsal roots and sensory neurons of the lumbar DRG. **a** To investigate whether the nociceptive impairment observed after intramuscular PFF-inoculation is due to the trans-synaptic propagation of α-Syn aggregates within the sensory nervous system, we performed immunodetection of PD-like α-Syn pathology in lumbar DRG and their dorsal sensory roots, spinal cord (dorsal horn and ventral horn), and brain (mesencephalic PAG and thalamic nuclei) in the vehicle, monomeric α-Syn- and PFF-injected M83^+/−^ cohorts, at 45 dpi. Illustration was created with BioRender.com. **b** IHC panel showing pathological pSer129-α-Syn immunoreactivity in axons of the sensory roots (upper right panel, yellow arrows) and neuron cell bodies of the lumbar L4 DRG (lower right pane, white arrows), in PFF-injected M83 mice at 45 dpi. pSer129-α-Syn was not detected in the PBS- or monomeric α-Syn- injected cohorts. pSer129-α-Syn is represented in green, neurofilament M (NF-M) as an axonal/neuronal marker in red and nuclei are labeled in blue with Hoechst. 20 × magnification. Scale bar = 40 µm; *n* = 4 mice per group. **c** Lumbar DRG (L3–L5) homogenates from PBS- (blue, *n* = 6), monomeric- (green, *n* = 6) and PFF-injected (red, *n* = 5) were separated by SDS-PAGE and visualized by immunoblotting with anti-α-Syn antibody, anti-pSer129-α-Syn, anti-β-actin, and anti-β-III tubulin. **d** Densitometry quantification of immunoblots as a ratio of pSer129/total α-Syn. Results shown as mean ± SEM as determined by one-way ANOVA followed by Tukey’s multiple comparison test. *P < 0.05

The DRG contain groups of pseudounipolar neurons surrounded by glial cells, comprising the greatest proportion of the body's peripheral sensory neurons which are responsible for the transduction of somatosensory and nociceptive input from the periphery to the CNS [33]. As the dorsal root exits the DRG to enter the spinal dorsal horn, sensory information ascends through central projections in the spinal cord and relays to the relevant regions of the brainstem and ventroposterior nuclei in thalamus [33]. To investigate whether the nociceptive impairment observed in the intramuscularly PFF-injected cohort is due to the prion-like spreading of α-Syn within the sensory nervous system, we performed immunofluorescence detection of PD-like α-Syn pathology (pSer129) in lumbar DRG and their dorsal sensory roots, spinal cord, and brain (mesencephalic PAG and thalamic nuclei) in the vehicle-, monomeric α-Syn- and PFF-injected M83^+/−^ cohorts, at 45 dpi, as illustrated in Fig. 3a.

Our results showed pSer129 immunofluorescence co-localization with the neuron-specific protein marker NF-M (Fig. 3b), in both DRG and dorsal roots of PFF-injected mice, indicating propagation of α-Syn aggregates through DRG neuron cell body and the axoplasm of the sensory roots. No pSer129 α-Syn immunostaining was found in these tissues from the PBS or monomeric α-Syn injected cohorts (Fig. 3b, left and central panels, respectively). Western blot analysis of lumbar DRG (L3–L5) homogenates further corroborated these observations, with the PFF-injected cohort presenting significantly increased levels of pathological pSer129, compared to PBS-or monomeric α-Syn- injected mice (Fig. 3c, d). Additionally, immunodetection against pSer129 in lumbar DRG (L3–L5) following unilateral intramuscular injection of PFF showed a significant increase of α-Syn insult in the ipsilateral DRG as compared to the contralateral (Additional file 1: Supplementary Fig. 2a, b) at 45 dpi, suggesting that any spread from ipsilateral to contralateral DRG is modest at this time point.

**Fig. 4:**
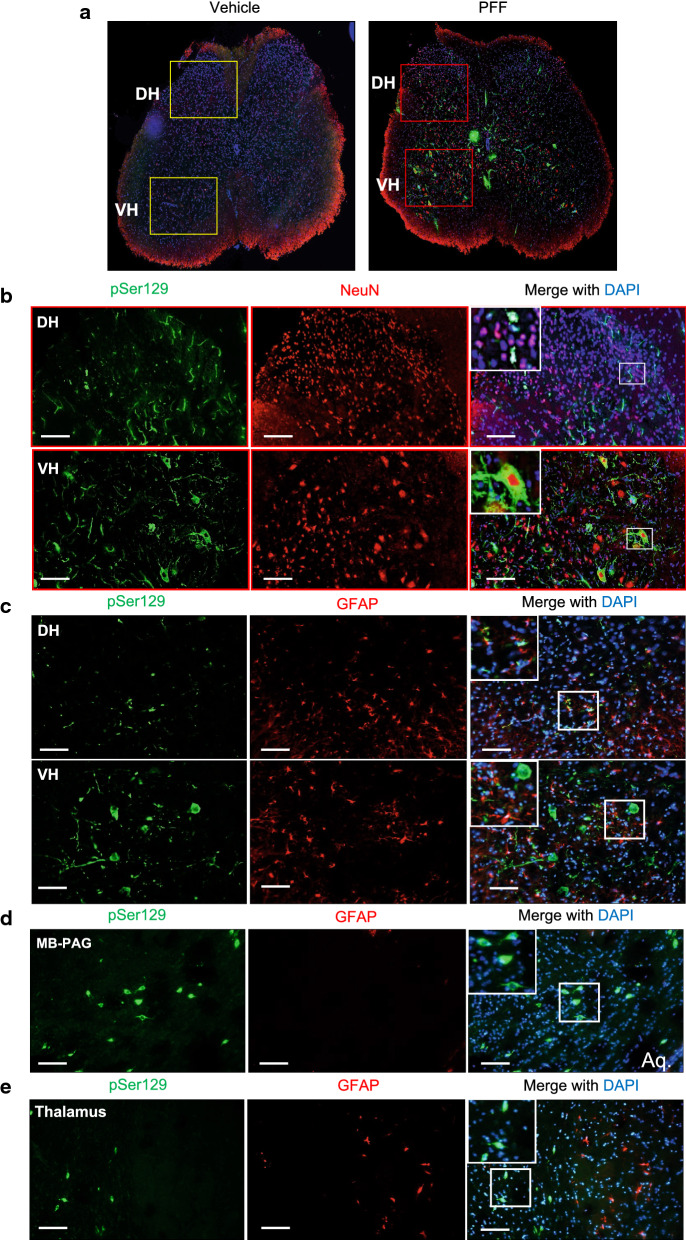
Intramuscular injection of α-Syn PFF prompt pathological aggregation of α-Syn in neurons of the lumbar spinal cord, midbrain periaqueductal grey and thalamus. **a** Panoramic views: pSer129-α-Syn (in green) co-detection with neuronal nuclei antigen (NeuN, in red) in vehicle- (PBS, pH 7.4, left panel) and PFF-injected mice (right panel) lumbar spinal cord sections at 45 dpi (DH, dorsal horn; VH, ventral horn), **b** pSer129-α-Syn (in green) co-detection with neuronal nuclei antigen (NeuN, in red) in α-Syn PFF- injected mice. **c** Astrogliosis in the lumbar spinal cord, midbrain periaqueductal grey and thalamus was observed in the PFF-injected cohort. pSer129-α-Syn (in green) and glial fibrillary acidic protein marker (GFAP, in red) immunoreactivity, VH and DH of lumbar spinal cord, **d** midbrain periaqueductual grey (MB-PAG) and **e** thalamus. DAPI (blue) was used to stain the nuclei. Scale bar = 100 µm; insets in merge show 63X magnified views

The pain processing neurons in DRG receive C and Aδ nociceptive and thermal inputs from peripheral receptors, and centrally project to the neurons in lamina I and II of the spinal cord [30]. The neurons in spinal lamina I (also known as the posteromarginal nucleus) transmit the nociceptive and thermal information into higher brain centers via spinothalamic, spinoreticular and spinomesencephalic projections [30]. Whereas, the neurons in lamina II (substantia gelatinosa) are mostly interneurons that send collaterals locally in the spinal laminae III-V. Therefore, we wanted to assess if the defective nociception in bilaterally PFF-injected mice (Fig. 2d, e) is associated with α-Syn pathology in central pathways of pain processing and/or modulation. Our immunohistochemistry (IHC) analysis showed robust α-Syn pathology (pSer129) in the spinal dorsal horn lamina I-V of the PFF- injected mice (Fig. 4a, b) but not vehicle-injected (Additional file 1: Supplementary Fig. 3a). Substantial α-Syn pathology was also detected in the spinal motor neurons of the ventral horn (lamina IX, Fig. 4a, b), as has been previously reported [47]. In contrast, no α-Syn pathology (pSer129) was observed in the control mice (Fig. 4a left panel, and Additional file 1: Supplementary Fig. 3a). Collectively, these sets of observations are consistent with a retrograde prion-like propagation of α-Syn aggregates via afferent sensory axons and ventral efferent motor spinal nerves.

Concomitant with accumulation of pSer129-positive neuronal inclusions, increased immunodetection of glial fibrillary acidic protein (GFAP), an astrogliosis marker, in the dorsal and ventral horns of the lumbar spinal cord was also seen (Fig. 4c, Additional file 1: Supplementary Fig. 4). Moreover, our data also reveal a remarkable extent of pSer129 burden in higher brain centers of pain processing and/or modulation including mesencephalic PAG (Fig. 4d) and thalamic nuclei (Fig. 4e) in PFF-injected mice 45 dpi, alongside with astrogliosis at the level of the lumbar spinal cord. Once again, no α-Syn pathology was identified in the spinal cord nor the analyzed brain areas of the vehicle injected mice (Additional file 1: Supplementary Fig. 3 ). To further establish the relevance of spinal α-Syn pathology (Fig. 4) to the defective nociception at 45 dpi (Fig. 2d, e), we also performed immunofluorescence analysis of the spinal cord at earlier time points (14 and 21 dpi, Additional file 1: Supplementary Fig. 5a, b). Our results show that, at these intervals, sparse pSer129 inclusions were detected in the dorsal horn (lamina I-V), being mostly present in the intermediate grey matter and ventral horn motor neurons (Additional file 1: Supplementary Fig. 5a, b).

### α-Syn-dependent morphological abnormalities in the peripheral sensory system

**Fig. 5:**
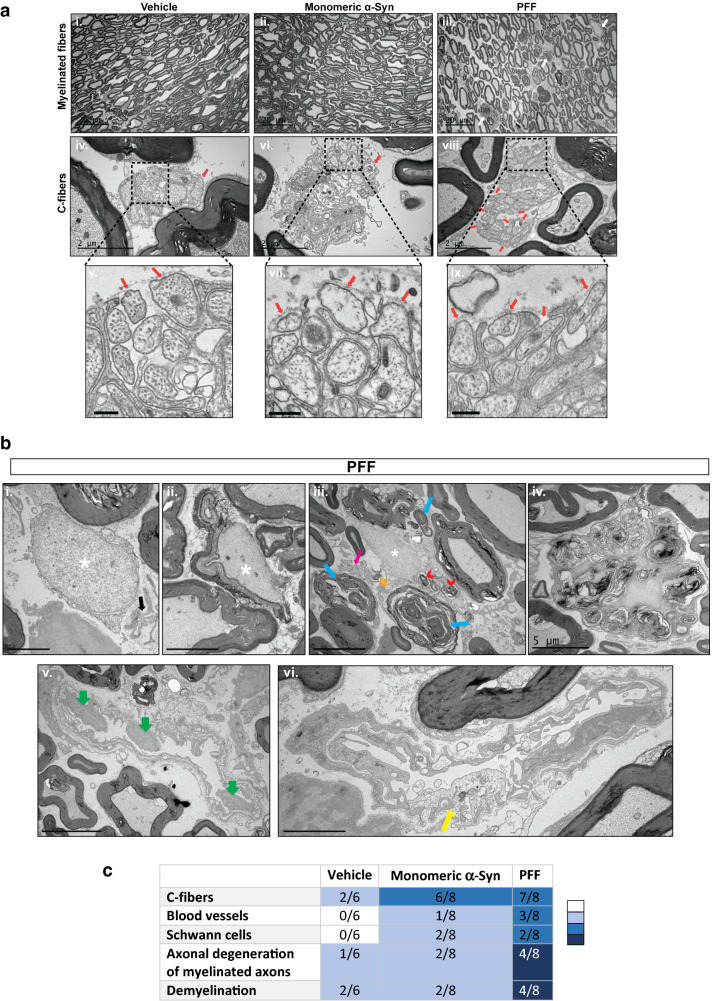
Peripherally injected PFF induces small- and medium-sized myelinated fiber pathology in the dorsal roots. **a** Morphological evaluation of the dorsal sensory roots by EM. Representative low magnification images of the sensory roots from vehicle- (panel i, PBS, pH 7.4), monomeric α-Syn (panel ii) and α-Syn PFF-injected mice (panel iii), denoting normal distribution of myelinated fibers with some morphological abnormalities identified in the PFF cohort (panel iii, white arrows). Scale bar 20 μm. Panels iv–ix denote high magnification pictures (scale bar = 2 µm), and respective insets (scale bar = 250 nm), showing an abnormal structure of the Remak bundles composed of numerous unmyelinated axons enclosed by basement membrane of Schwann cells, particularly in the monomeric and PFF- injected mice (panel vi–ix). Note electron loose Schwann cell processes, and the lack of these processes around some of the axons (orange arrows). **b** Image displaying completely (panel i) or partially demyelinated axon (panel ii) (> 1.5 μm diameter; white asterisks). In Panel (i), flat sheets of Schwann cell cytoplasmic processes enclosed by a common basement membrane, designated as bands of Büngner (black arrow) that are usually formed after degeneration of unmyelinated axons, can also be observed. Panel (iii) Picture showing degeneration of small- and medium-sized myelinated fibers (blue arrows), Schwann cell cytoplasm containing degradation products and myelin ovoids (red arrowhead), free debris in the endoneurium (orange arrow) and disorganized Remak bundles with few unmyelinated C-fibers (pink arrow). Panel (iv) A macrophage engulfing myelin fragments is highlighted. Panel (v) Green arrows feature projection of endothelial cell nuclei into the lumen of blood vessels. Panel (vi) Exhibits an endothelial cell with abnormal morphology, with irregular small branches and swollen electron loose cytoplasm (yellow arrow). Scale bars are 2 μm (Panels i, vi) and 5 μm (Panels ii, iii, iv, v). **c** Dorsal sensory roots semi-quantitative pathology scoring summary of the three cohorts in this study; the number of mice with detectable pathological abnormalities in C-fibers, blood vessels, Schwan cells or axonal degeneration and demyelination in the endoneurium (n/6 for vehicle- injected mice and n/8 for both monomeric α-Syn- and PFF-injected mice) is shown along with density of pathology represented by colour where darker colour indicates aggravated pathology

Dorsal sensory roots were analyzed by transmission electron microscopy (TEM) and, as expected, myelinated fibers with different range of diameters were observed (Fig. 5a panels i–iii), with C-fibers distributed throughout the endoneurium. Strikingly, C-fibers were abnormal in all the examined groups, more severely in monomeric α-Syn- and PFF- injected groups, with some axons displaying a large gap between the axoplasm and the Schwann cell cytoplasm, conferring an abnormal structure to the Remak bundle with semi-naked unmyelinated axons that are only in contact with the Schwann cell basal lamina (Fig. 5a panels vi–ix). Furthermore, the occurrence of unmyelinated axons with intact axolemma but darker axoplasm suggests ongoing axonal degeneration (Fig. 5a panels vi, viii). The fact that these pathological alterations were found in all cohorts, including the PBS- injected mice, suggests that overexpression of α-Syn A53T human mutant might be associated with abnormal C-fiber morphology in the dorsal roots. Other than abnormal C-fiber morphology, sensory roots from PBS- and monomeric α-Syn- injected cohorts were generally normal, with a few samples showing myelin degradation (Fig. 5c). Completely (Fig. 5b, panel i) or partially demyelinated axons (Fig. 5b, panel ii) were observed in the endoneurium, particularly in the PFF cohort (Fig. 5c).

Besides the abnormal appearance of Schwann cells in the Remak bundles described above, additional mild Schwann cell pathology was found in 2 animals injected with PFF, with Schwann cell cytoplasmic extensions abundantly present in the endoneurium, resembling bands of Büngner (Fig. 5b–i), an indication of nerve injury and neurodegeneration [28, 53]. In addition, 4 out of 8 of the PFF-inoculated cohort showed extensive axonal degeneration together with secondary demyelination and axonal atrophy, mainly in the small and medium myelinated fibers (Fig. 5b, panel iii). The degradation rest products were observed extracellularly in the endoneurium (Fig. 5b, panel iii), where some macrophages were also found, engulfing myelin debris (Fig. 5b, panel iv).

Indication of blood vessel damage was evident in the PFF cohort, with 3 out of 8 mice showing endothelial lumen invasion (Fig. 5b, panel v) and abnormal endothelial cell morphology with irregular small branches (Fig. 5b, panel vi). In contrast, none or 1 out of 8 samples, for PBS- and monomer- injected groups, respectively, presented an abnormal blood vessel (Fig. 5c), with the nucleus of an endothelial cell projecting into the lumen.

Overall, all of the ultrastructural abnormalities observed in the sensory afferents of the PFF- cohort support a α-Syn aggregation-dependent degeneration of axons and myelin resulting in impaired sensory processing and development of mechanical allodynia and reduced nerve conduction velocities.

## Discussion

A substantial proportion of PD patients (up to 85%) present some form of acute or chronic pain, including visceral, neuropathic, musculoskeletal or dystonic pain [15, 61], frequently preceding motor disturbances [43]. Although chronic pain in PD is a recurrent and important non-motor symptom, it is often overlooked as a pre-diagnostic presentation or early symptom, with tendency for being even commonly misdiagnosed and treated as depression, shoulder pain or muscle stiffness [49]. The various pain presentations of PD have been extensively characterized and categorized [5, 57], but the underlying mechanisms remain elusive and poorly understood. Currently, there are no proven effective pharmacological therapies to specifically alleviate PD-associated pain [57]. Therefore, a better understanding of the pathophysiological mechanisms of PD-associated pain, and characterization of appropriate PD animal models, is a prerequisite for the design of innovative treatments able to address this critical issue. Existing animal models for PD, including acute pharmacological (reserpine and haloperidol) or toxin-based (MPTP, rotenone and 6-OHDA) mouse models have been used to study PD-induced pain [8]. Although these models present some level of face and predictive validity for the understanding of the pathophysiology of pain symptoms in PD, they have major shortcomings. These include the absence of Lewy body-like pathology, invasive routes of administration (intraventricular, intracisternal, intracerebral) provoking blood–brain barrier disruption and edema, high toxicity, low reproducibility, or limited time window for the investigation of the disease progression and potential therapeutical interventions [6, 8].

In this study, we show that retrograde templated spreading of pathological α-Syn is associated to an aggregate-dependent sensory nerve degeneration and defective nociception. The results presented here demonstrate that peripherally injected PFF in the gastrocnemius muscle of M83^+/−^ mice, induce α-Syn pathology in sensory neurons and dorsal roots, and propagates into the CNS throughout neuroanatomical connected pathways, while modulating nociceptive pain responses. Therefore, these data highlight the potential of this mouse model as a valuable tool for future research on the molecular mechanisms involved in PD-associated pain.

The mouse sciatic nerve (about 70%) consists of myelinated and unmyelinated sensory axons, predominantly from L3–L5 DRG and is the major nerve supply to the hindlimb [58]. We have found that accumulation of α-Syn aggregates in the afferent sensory system (including sensory neurons of the DRG, axons from the dorsal roots and spinal dorsal horn neurons, lamina I and II) occurs concomitantly with the emergence of defective nociception. Although α-Syn pathology in the spinal cord has not been extensively studied, there is evidence that some lamina I neurons in the thoracic segment of PD patients harbor α-Syn inclusions [7]. Nevertheless, we here observed that pSer129 pathology in the spinal cord manifests first in laminae VIII-IX of the ventral horn, comprising motor neurons whose axons innervate mostly striated or skeletal muscle fibers [30]. This may indicate preferential spreading of α-Syn pathology via myelinated motor axons [3].

We also detected substantial α-Syn pathology in the mesencephalic PAG and thalamic nuclei, which are major relay centres for pain information in CNS. While the thalamic nuclei are the major gateway to the somatosensory cortex, PAG contains neurons that participate in the regulation of autonomic function, pain modulation and circadian rhythms due to their widespread connectivity in brainstem and hypothalamus [37]. In this context, α-Syn pathology is also reported in PAG in neuropathological studies in PD and related diseases [27, 50], further supporting the relevance of this peripheral-to-central rodent model of templated propagation of α-Syn aggregates.

Moreover, PFF-inoculation elicited GFAP immunoreactivity that mirrored the spatiotemporal accumulation of pS129-α-Syn in the dorsal and ventral horns of the spinal cord, suggesting aberrant tissue homeostasis caused by the propagation of α-Syn aggregate seeds along interconnected neuronal populations from the PNS to the CNS. In addition, mice receiving a unilateral intramuscular injection of PFF showed significantly higher levels of pSer129 pathology in the ipsilateral lumbar DRGs compared to the contralateral, supporting rostral propagation of α-Syn pathology through direct neuroanatomical connections [3].

Intramuscular injection of PFF elicited hindlimb clasping behaviour, an abnormal phenotype also observed in mice with lesions in cerebellum, basal ganglia, and neocortex [35], as well as in transgenic mice with amyloid pathology and neurodegeneration [34, 35]. In addition, the PFF-injected cohort presented impaired mechanical allodynia, i.e. a reduced nociceptive threshold to a non-noxious stimuli, in a similar fashion to what is clinically observed in PD patients [26, 56]. Damage of medium and small myelinated and unmyelinated fibers in the dorsal sensory roots of PFF-inoculated animals might underlie the observed pain-like phenotype. These results further support the involvement of pathological α-Syn propagation regarding sensory abnormalities related to nociceptive processing.

Peripheral neuropathy typically starts from an asymptomatic subclinical stage, in which neurological changes have already begun but can only be detected through more quantitative and sensitive testing. Although there is no “gold standard” diagnostic methodology for evaluating early stages of peripheral neuropathy, electrophysiological nerve conduction velocity studies are widely accepted as a precise and reliable method of classification and quantification of peripheral nerve damage [42]. While performing nerve conduction studies to investigate peripheral nerve damage in our PD mouse model, we found a discrete but significant reduction of sensory and motor nerve conduction velocities in the PFF-injected mice, equivalent to what is reported in idiopathic PD patients, including those under treatment with L-Dopa [38, 55], and also seen in other mouse models of peripheral sensory neuropathies [21]. The presence of peripheral neuropathy as demonstrated by pS129-a-syn in skin is frequent in PD and even presymptomatic PD as demonstrated in REM sleep behavior disorder patients [16].

Degeneration of the small and medium myelinated fibers in the PFF cohort may explain the observed reduction in conduction velocity. Other ultrastructural deficits found by EM might additionally have an impact on these electrophysiological parameters, such as the abnormal endothelial cell morphology and invasion of the blood vessel lumen, which in a longer term could lead to endothelial and microvascular dysfunction, affecting the endoneurial nutritive blood flow and thus compromising signal conduction [11, 22, 45]. In the future, it would be interesting and relevant to explore whether aggregation and spreading of α-Syn in the PNS might have an impact on the distribution of sodium channels in the nodes, internodal distances [2, 59], the function of the Na/K ATPase [60], density of type I muscle fibers [4] as well as the mitochondrial content in the fibers [39], as all of these functional cellular mechanisms or parameters can impact the nerve conduction velocities.

The altered morphology of the Remak bundles together with the frequent observed signs of ultrastructural damage, such as small and medium myelinated fiber degeneration and the presence of flat sheets of Schwann cell cytoplasmic processes (bands of Büngner) in the sensory dorsal roots, can also also contribute to the observed reduction in nerve conduction velocities as well as to the increased mechanical allodynia in the PFF mice, as compared with the control counterparts.

Although our results support the face and predictive validity of the intramuscular PFF injection model for a better understanding of the pathophysiology underlying pain in PD, and provide a testbed for development of modulation therapies, there are also limitations that should be taken into account. For instance, the distribution of α-Syn pathology and onset of disease varies on the PFF injection site (e.g. muscle, olfactory bulb, brain, gut), the amount and type of PFF, and the animal species and lines [9].

In summary, we have here provided conclusive evidence that peripheral seeding with α-Syn fibrils in transgenic M83^+/−^ mice results in a robust trans-synaptic α-Syn spreading of endogenously recruited α-Syn through interconnected neurons in a prion-like manner. The propagation of α-Syn pathology from the peripheral sensory system to the CNS was concomitant with sensory nerve degeneration and nociceptive hypersensitivity. We hypothesize that these dysfunctions might contribute to the multifaceted etiology and symptomatology of pain in PD. Furthermore, our observations support the face and predictive validity of our rodent model for a better understanding of the molecular mechanisms underlying pain in PD and provide a testbed for development of innovative therapeutic approaches for the treatment of this common debilitating non-motor symptom.

## Supplementary Information

**Additional file 1.** **Fig. S1**. Seeding and staining controls for OHCS experiment. a, b) C57BL/6J OHCS injected with sterile PBS (**a**) or monomeric α-Syn (b) do not show any aggregation either at injection site (DG) or elsewhere at 7 dpi, as detected by conformation-specific MJF-14 (green) and pSer129 (11A5, red). Scale bars = 20 μm (**a**), 100 μm (**b**).** c**) Injection of PFF in α-Syn knockout OHCS does not result in any aggregate pathology at 7 dpi, due to the lack of endogenous α-Syn needed to template aggregation. Conformation-specific MJF-14 (green) was used to detect pathology. Scale bar = 100 μm. **Fig. S2**. Unilateral intramuscular injection of PFF elicits higher pSer129 insult in the ipsilateral lumbar DRG compared to the contralateral side. Threemonth-old TgM83+/− mice were injected with full-length (1-140) mouse α-Syn PFF (*n*=4) in the right hindlimb (ipsilateral, in red) or vehicle in the left hindlimb (PBS pH 7.4, contralateral, in blue). **a**) Proteins from lumbar DRG (L3-L5) homogenates were separated by SDS-PAGE and visualized by immunoblotting with anti-α-Syn antibody, anti-pSer129-α-Syn, anti-b-actin, and anti-b-III tubulin. **b**) Densitometry quantification of immunoblots as a ratio of pSer129/total α-Syn. Results are shown as mean ± SEM, as determined by one-way ANOVA followed by Tukey’s multiple comparison test. *P < 0.05. **Fig. S3**. Immunofluorescence detection of astrogliosis in relation to pSer129-α-Syn pathology in lumbar spinal cord, midbrain periaqueductal grey and thalamus of vehicle- injected M83 mice. **a**) pSer129-α-Syn(in green) co-detection with neuronal nuclei antigen (NeuN, in red) in vehicle-injected mice in dorsal (DH) and ventral (VH) and horn of lumbar spinal cord. **b**) pSer129-α-Syn (in green) and glial fibrillary acidic protein marker (GFAP, in red) immunoreactivity in DH and VH of lumbar spinal cord, **c**) midbrain periaqueductual grey (MB-PAG) and **d**) thalamus. DAPI (blue) was used to stain the nuclei. Scale bar = 100 μm; insets in merge show 63X magnified views. **Fig. S4**. Immunofluorescence quantification of astrogliosis in lumbar and ventral horn of the spinal cord, midbrain periaqueductal grey (PAG) and thalamus in vehicle and PFF- injected M83 mice. Quantification was performed on 10X views using Zen software (Zeiss). DAPI was used as a cell marker, GFAP quantitation is expressed as GFAP+ cells/mm2 in the indicated regions. Results shown as mean ± SEM as determined by ordinary one-way ANOVA followed by multiple comparison test. ***P < 0.001; ****P < 0.0001. VH, ventral horn, DH, dorsal horn, PAG, periaqueductal grey, thalamus ventroposterior. **Fig. S5**. Immunofluorescence for pSer129-α-Syn pathology in lumbar spinal cord of PFF- injected M83 mice. pSer129-α-Syn immunoreactivity (in green) in the dorsal (DH) and ventral (VH) horns, and the intermediate grey (IG), with 20X magnified views of grey matter (red rectangles) and white matter (yellow rectangles) as overlayimages at **a**) at 14 dpi and **b**) 21 dpi. I-X represent Rexed laminae; CC, central canal. DAPI (blue) was used to stain the nuclei.Scale bar 100 μm.

## Data Availability

The data that supports the findings of this study are available from the corresponding authors upon reasonable request.
