# Supplementary material for: Trans-synaptic spreading of alpha-synuclein pathology through sensory afferents leads to sensory nerve degeneration and neuropathic pain
Source: Acta Neuropathol Commun. 2021 Feb 25;9:31. doi: 10.1186/s40478-021-01131-8 (PMC7905893; doi:10.1186/s40478-021-01131-8)
Supplement: Supplementary file 1 — Additional file 1. Fig. S1. Seeding and staining controls for OHCS experiment. a, b) C57BL/6J OHCS injected with sterile PBS (a) or monomeric α-Syn (b) do not show any aggregation either at injection site (DG) or elsewhere at 7 dpi, as detected by conformation-specific MJF-14 (green) and pSer129 (11A5, red). Scale bars = 20 μm (a), 100 μm (b). c) Injection of PFF in α-Syn knockout OHCS does not result in any aggregate pathology at 7 dpi, due to the lack of endogenous α-Syn needed to template aggregation. Conformation-specific MJF-14 (green) was used to detect pathology. Scale bar = 100 μm. Fig. S2. Unilateral intramuscular injection of PFF elicits higher pSer129 insult in the ipsilateral lumbar DRG compared to the contralateral side. Threemonth-old TgM83+/− mice were injected with full-length (1-140) mouse α-Syn PFF (n=4) in the right hindlimb (ipsilateral, in red) or vehicle in the left hindlimb (PBS pH 7.4, contralateral, in blue). a) Proteins from lumbar DRG (L3-L5) homogenates were separated by SDS-PAGE and visualized by immunoblotting with anti-α-Syn antibody, anti-pSer129-α-Syn, anti-b-actin, and anti-b-III tubulin. b) Densitometry quantification of immunoblots as a ratio of pSer129/total α-Syn. Results are shown as mean ± SEM, as determined by one-way ANOVA followed by Tukey’s multiple comparison test. *P < 0.05. Fig. S3. Immunofluorescence detection of astrogliosis in relation to pSer129-α-Syn pathology in lumbar spinal cord, midbrain periaqueductal grey and thalamus of vehicle- injected M83 mice. a) pSer129-α-Syn(in green) co-detection with neuronal nuclei antigen (NeuN, in red) in vehicle-injected mice in dorsal (DH) and ventral (VH) and horn of lumbar spinal cord. b) pSer129-α-Syn (in green) and glial fibrillary acidic protein marker (GFAP, in red) immunoreactivity in DH and VH of lumbar spinal cord, c) midbrain periaqueductual grey (MB-PAG) and d) thalamus. DAPI (blue) was used to stain the nuclei. Scale bar = 100 μm; insets in merge show 63X magnified v [file 40478_2021_1131_MOESM1_ESM.pdf]

Electronic Supplementary Material for:

Trans-synaptic spreading of alpha-synuclein pathology through sensory afferents leads to sensory nerve degeneration and neuropathic pain

Nelson Ferreira[1#*], Nádia Pereira Gonçalves[1#*], Asad Jan[1], Nanna Møller Jensen[1], Amelia Van Der Laan[1], Simin Mohseni[2], Christian Bjerggaard Vægter[1], Poul Henning Jensen[1*]

[1] Danish Research Institute of Translational Neuroscience (DANDRITE), Nordic EMBL Partnership for Molecular Medicine, Department of Biomedicine, Aarhus University, 8000 Aarhus C, Denmark.
[2] Department of Biomedical and Clinical Sciences, Linköping University, Linköping, Sweden.

[#] These authors contributed equally to this work.
[*] To whom the correspondence should be addressed. Email: nelson@biomed.au.dk , npg@dandrite.au.dk and phj@biomed.au.dk

Supplementary Fig. 1: Seeding and staining controls for OHCS experiment. a, b) C57BL/6J OHCS injected with sterile PBS (a) or monomeric α-Syn (b) do not show any aggregation either at injection site (DG) or otherwise at 7 dpi, as detected by conformation-specific MJF-14 (green) and pSer129 (11A5, red). Scale bars = 20 µm (a), 100 µm (b). **c)** Injection of PFF in α-Syn knockout OHCS does not result in any aggregate pathology at 7 dpi, due to the lack of endogenous α-Syn needed to template aggregation. Conformation-specific MJF-14 (green) was used to detect pathology. Scale bar = 100 µm.

Supplementary Fig 2. Unilateral intramuscular injection of PFF elicits higher pSer129 insult in the ipsilateral lumbar DRG compared to the contralateral side. Three-month-old TgM83$^{+/-}$ mice were injected with full-length (1-140) mouse α-Syn PFF (*n*=4) in the right hindlimb (ipsilateral, in red) or vehicle in the left hindlimb (PBS pH 7.4, contralateral, in blue). **a)** Proteins from lumbar DRG (L3-L5) homogenates were separated by SDS-PAGE and visualized by immunoblotting with anti-α-Syn antibody, anti-pSer129-α-Syn, anti-β-actin, and anti-β-III tubulin. **b)** Densitometry quantification of immunoblots as a ratio of pSer129/total α-Syn. Results are shown as mean ± SEM, as determined by one-way ANOVA followed by Tukey's multiple comparison test. *$P < 0.05$.

Supplementary Fig 3. Immunofluorescence detection of astrogliosis in relation to pSer129-α-Syn pathology in lumbar spinal cord, midbrain periaqueductal grey and thalamus of vehicle-injected M83 mice. a) pSer129-α-Syn (in green) co-detection with neuronal nuclei antigen (NeuN, in red) in vehicle-injected mice in dorsal (DH) and ventral (VH) and horn of lumbar spinal cord. **b)** pSer129-α-Syn (in green) and glial fibrillary acidic protein marker (GFAP, in red) immunoreactivity in DH and VH of lumbar spinal cord, **c)** midbrain periaqueductual grey (MB-PAG) and **d)** thalamus. DAPI (blue) was used to stain the nuclei. Scale bar = 100 µm; insets in merge show 63X magnified views.

Supplementary Fig. 4. Immunofluorescence quantification of astrogliosis in lumbar and ventral horn of the spinal cord, midbrain periaqueductal grey (PAG) and thalamus in vehicle and PFF-injected M83 mice. Quantification was performed on 10X views using Zen software (Zeiss). DAPI was used as a cell marker, GFAP quantitation is expressed as GFAP^{+} cells/mm^{2} in the indicated regions. Results shown as mean ± SEM as determined by ordinary one-way ANOVA followed by multiple comparison test. ***$P < 0.001$; ****$P < 0.0001$. VH, ventral horn, DH, dorsal horn, PAG, periaqueductal grey, thalamus ventroposterior.

Supplementary Fig. 5. Immunofluorescence for pSer129-α-Syn pathology in lumbar spinal cord of PFF- injected M83 mice. pSer129-α-Syn immunoreactivity (in green) in the dorsal (DH) and ventral (VH) horns, and the intermediate grey (IG), with 20X magnified views of grey matter (red rectangles) and white matter (yellow rectangles) as overlay images at **a**) at 14 dpi and **b)** 21 dpi. I-X represent Rexed laminae; CC, central canal. DAPI (blue) was used to stain the nuclei. Scale bar 100 μm.
